# Effects of Hair Metals on Body Weight in Iranian Children Aged 20 to 36 Months

**Published:** 2017-08

**Authors:** Mohsen VIGEH, Kazuhito YOKOYAMA, Takehisa MATSUKAWA, Atsuko SHINOHARA, Mamak SHARIAT, Katsumi OHTANI

**Affiliations:** 1. Maternal, Fetal & Neonatal Research Center, Imam Khomeini Hospital, Tehran University of Medical Sciences, Tehran, Iran; 2. Dept. of Epidemiology and Environmental Health, Faculty of Medicine, Juntendo University, 2-1-1 Hongo, Bunkyo-Ku, Tokyo, Japan; 3. Occupational Epidemiology Research Group, National Institute of Occupational Safety and Health, 6-21-6 Nagao, Tama-Ku, Kawasaki, Japan

**Keywords:** Metal, Children, Gender, Growth, Weight, Hair

## Abstract

**Background:**

Although the level of exposure to many toxic metals decreased recently, the adverse effects of these metals on children’s growth and development remain a serious public health issue.

**Methods:**

The present study was conducted in three teaching hospitals affiliated with Tehran University of Medical Sciences (Tehran, Iran) from Sep 2012 to Mar 2013. To study the relationship between metals and childhood growth, concentrations of zinc and several potentially toxic metals (lead, cadmium, antimony, cobalt, and molybdenum) were measured in scalp hair for 174 children, aged 20 to 36 months.

**Results:**

The hair concentrations of cobalt were significantly (*P*<0.05) higher in children at the lower percentile of weight than in higher-weight children (0.026 ± 0.04 vs. 0.015 ± 0.01 μg/g, respectively). Hair contents of lead, cobalt, and antimony were significantly higher (*P*<0.05) in girls than in boys (8.08 ± 8.7 vs. 4.92 ± 5.6 μg/g for lead, 0.026 ± 0.03 vs. 0.16 ± 0.02 μg/g for cobalt, and 0.188 ± 0.29 vs. 0.102 ± 0.12 μg/g for antimony). There were also significant correlations between lead and other metals in the children’s hair.

**Conclusion:**

Gender may play a significant role in absorption and/or accumulation of metals. It should be considered when we study metal toxicity in children.

## Introduction

Childhood exposure to toxic metals is a serious public health problem in many developing countries (1) and usually, involves exposure to a mixture rather than individual metals (2). Thus, it is critical to investigate metals interactions due to mixed metals exposure has been reported to increase the risk of adverse effects on children’s growth and development (3). However, few epidemiological surveys have studied multiple metals exposure and their interaction in children.

Personal characteristics, such as age and sex, have been reported to influence metals’ metabolisms, which may induce metal toxicities (4–9).

This may occur via synergism in the production of reactive oxygen species, cytokine pathway activation, and cytochrome P450 enzyme activity (5). For instance, glutathione (an antioxidant agent) plays a central role against biotic stress in coping with different metals (6, 7), especially when a multiple metal exposure occurs (8). Epidemiological (9, 10) and experimental (rats) (11) studies have shown significant differences in levels of glutathione and superoxide dismutase between female and male subjects, which may lead to differing in protection against oxidative stress (12).

Studies on preschool children found considerable numbers of underweight children (7.5% to 20%) (13–16) and children of short stature (6.6%) (13) in Iran compared to international data (17), with a significant difference between boys and girls (16). The reasons for Iranian children growth problem is not fully understood yet. Although socioeconomic status and subsequent diets insufficiency may play an important role, other factors, such as genes, gender, and environmental pollution, may be involved as well. Iranian infants are exposed to relatively high levels of metals from industrial activities, via polluted air and soils (18, 19) or through breast milk (mean lead level of 2.4 μg/dL) (20). In addition, a recent study in Iran reported higher levels of metal in the first haircut of newborn than the mothers’ hair (157 vs 87 μg/kg for cadmium, 246 vs 198 μg/kg for mercury, 14313 vs 11776 μg/kg for copper, and 408207 vs 52022 μg/kg for aluminum, respectively) (21), which can suggest a free pass of these metals via umbilical cord to the fetuses.

Serial blood measurements may offer a better estimation of the body burden of metals than a single measurement, but they are usually impractical. Instead, measuring metals in hair samples offers advantages for routine clinical screening and diagnosis as an alternative to blood and urine sampling (22–24), which can present information on exposure over a long period of time and noninvasive sample collection.

The present study measured concentrations of metals (lead, zinc, cadmium, antimony, cobalt, and molybdenum) in children’s scalp hair to evaluate metal effects on children’s weight gain.

## Materials and Methods

### Study participants

The present study was conducted in three teaching hospitals affiliated with Tehran University of Medical Sciences (Tehran, Iran) from Sep 2012 to Mar 2013. The study included 174 children aged 20 months to 3 yr.

The Ethical Committees for the Vice-Chancellor of the Research Department and Institutional Review Board of Tehran University of Medical Sciences approved the study design and procedures. The study was conducted under the supervision of the Maternal, Fetal & Neonatal Research Center, Tehran University of Medical Sciences. The study purpose and procedures were fully explained to the children’s parents, and the study was conducted with their informed consent. Participation in the study was strictly voluntary.

### Questionnaire and measurements

Information about participants’ characteristics was gathered using a structured questionnaire developed for this study (see Appendix for main items). Anthropometric variables were obtained from growth chart records. Children were weighed when wearing underwear only, using a standard balance beam scale. With the children in standing position, height was measured to the nearest 0.1 cm using a rigid stadiometer tested for accuracy.

### Collection and analysis of hair samples

For removing external contamination from children’s hair, mothers were asked to wash the children’s hair with shampoo or soap, then rinse with plenty of tap water (at least five minutes) immediately prior to sampling. None used any type of hair treatment. After cutting children’s hair (about 3 cm from the child’s scalp; 2–5 g), the sample was placed in a plastic pack and transferred to the laboratory for metals analysis.

For analysis, hair samples were weighed (5–10 mg) and put into Teflon perfluoroalkoxy bottles, and 0.4 ml of concentrated nitric acid (ultrapure grade, Tama Chemicals Co., Kawasaki, Japan) was added. The bottles were left overnight at room temperature (18–28 °C). The sample mixture was then digested with 0.2 ml of hydrogen peroxide (ultrapure grade, Tama Chemicals Co., Kawasaki, Japan) using a microwave oven (MLS-1200 MEGA, Milestone Srl, Bergamo, Italy). This process was done in five steps using various power levels set at 250, 0, 400, 650, and 250 W for 6, 1, 6, 6, and 6 min, respectively. Next, the volume of the digested sample was adjusted to 1.0 ml using ultrapure water. After dilution with 0.5% nitric acid solution, subsequent measurements for metal concentrations were done by inductively coupled plasma mass spectrometry (ELAN 6000, PerkinElmer, Waltham, MA, USA) using multi-element standard solutions XSTC-13B and XSTC-622 (SPEX CertiPrep, Metuchen, NJ, USA). Each measurement was repeated three times, and the average of the three measurements was used for statistical analyses. For instrument calibration throughout the measurements, at least 10% of the analyses were external standards, and 5% were blank (pure water).

### Data analysis

Children weight gain (kg) was calculated by subtracting the children’s weight at 18 months old from their birth weight recorded on the growth chart. Pearson correlation coefficients were used to study relationships between metals. Student’s t-tests were calculated to investigate differences in metal concentrations between relatively high and low weight gain children (<50th and >50th percentile) at 18 months of age. The same statistical test was employed to compare metal concentrations between boys and girls. We employed ANOVA test to compare children’s hair levels of metals among the city districts. Multiple regression analysis was used to examine the relationships between metal levels and children’s weight, controlling for possible confounding variables (maternal age, weight, height, and child’s age and sex). Statistical analyses were performed using SPSS (IBM SPSS, Armonk, NY, USA), and statistical significance was determined using *P*<0.05.

## Results

Three children (two boys and one girl) weight were at less than percentile five and nine children (3 boys and 6 girls) weight were higher than percentile 95, according to the World Health Organization (WHO) classification for 18 months old children, separately for boys and girls. Student’s t-test showed that children with relatively lower weight at 18 months (<50th percentile) had significantly higher levels of cobalt in their hair than children with higher weight (mean ± SD 0.026 ± 0.04 and 0.015 ± 0.01 μg/g, respectively, *P*=0.021) (Table 1).

**Table 1:** Comparison of metal levels in children’s hair according to percentiles of children weight at 18 months old (n = 138) ^‡^

**Weight percentile**	**< 50 n= 70†**	**> 50 n = 68†**	***P*-value**
Hair level of (μg/g)			
Lead	6.77 ± 6.9	5.14 ± 4.9	NS
Zinc	122 ± 76	123 ± 58	NS
Cadmium	0.110 ± 0.14	0.08 7± .07	NS
Antimony	0.159 ± 0.24	0.110 ± 0.17	NS
Cobalt	0.026 ± 0.04	0.015 ± 0.01	< 0.021
Molybdenum	0.091 ± .05	0.099 ± .10	NS
Child age (month)	27.2 ± 5.0	29.0 ± 4.6	0.031
Maternal			
Age (yr)	25.1 ± 4.6	25.8 ± 4.1	NS
Weight (kg)	61.8 ± 12.8	65.1 ± 9.0	NS
Height (cm)	157.2 ± 7.1	160.2 ± 60.1	0.041

‡ .36 missing data (due to unreliable metal measurement according to standards and blank or, missed data on subjects’ characteristics)

† Mean ± SD; Student t-test

NS: none significant

There was not the same result for children’s weight percentiles at the birth, 6 and 12 months old. Multiple regression analysis showed no significant relationship between children’s weight and hair concentration of cobalt when adjusted for covariates (data not shown). Hair concentrations of lead, cobalt, and antimony were significantly higher in girls than in boys (8.08 ± 8.7 vs. 4.92 ± 5.6 μg/g for lead, 0.026 ± 0.03 vs. 0.16 ± 0.02 μg/g for cobalt, and 0.188 ± 0.29 vs. 0.102 ± 0.12 μg/g for antimony, respectively, *P*<0.05) (Table 2).

**Table 2:** Comparison of metal levels in children’s hair, weight, and height between boys and girls (n = 155)^‡^

	**Boys n = 74*^†^***	**Girls n = 81*^†^***	***P*-value**
Hair level of (μg/g)			
Lead	4.92 ± 5.6	8.08 ± 8.7	0.008
Zinc	124 ± 56	117 ± 72	NS
Cadmium	.086 ± .09	.118 ± .12	NS
Antimony	.102 ± .12	.188 ± .29	0.017
Cobalt	.016 ± .02	.026 ± .03	0.029
Molybdenum	.088 ± .05	.108 ± .10	NS
Child			
Age (month)	26.3 ± 7.1	27.1 ± 5.7	NS
Birth weight (kg)	4.11 ± 4.6	3.43 ± 3.0	NS
Birth height	50.6 ± 2.9	49.2 ± 3.0	0.003
Weight at 6 months (kg)	7.87 ± 0.8	7.3 ± 0.8	0.001
Height at 6 months (cm)	67.2 ± 3.2	65.5 ± 3.5	0.002
Weight at 12 months (kg)	9.8 ± 0.9	9.2 ± 0.9	0.001
Height at 12 months (cm)	75.2 ± 3.4	73.8 ± 3.0	0.002
Weight at 18 months (kg)	11.2 ± 1.1	10.5 ± 1.3	0.001
Height at 18 months (cm)	81.7 ± 3.3	80.1 ± 3.4	0.006
Weight gain (kg)	6.97 ± 4.9	7.04 ± 3.25	NS
Maternal			
Age (year)	25.7 ± 4.0	25.2 ± 4.5	NS
Weight (kg)	62.4 ± 10.5	65.0 ± 11.1	NS
Height (cm)	159.7 ± 6.7	158.1 ± 6.5	NS

‡ 19 missing data (due to unreliable metal measurement according to standards and blank or, missed data on subjects’ characteristics) //

† Mean ± SD; Student *t*-test //

NS: none significant

**Fig. 1:**
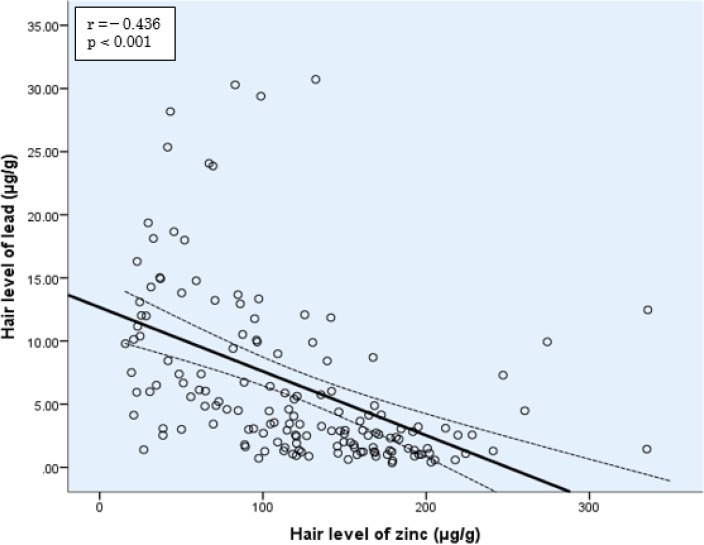
Pearson correlation between concentrations of lead and zinc in children hair

**Fig.2:**
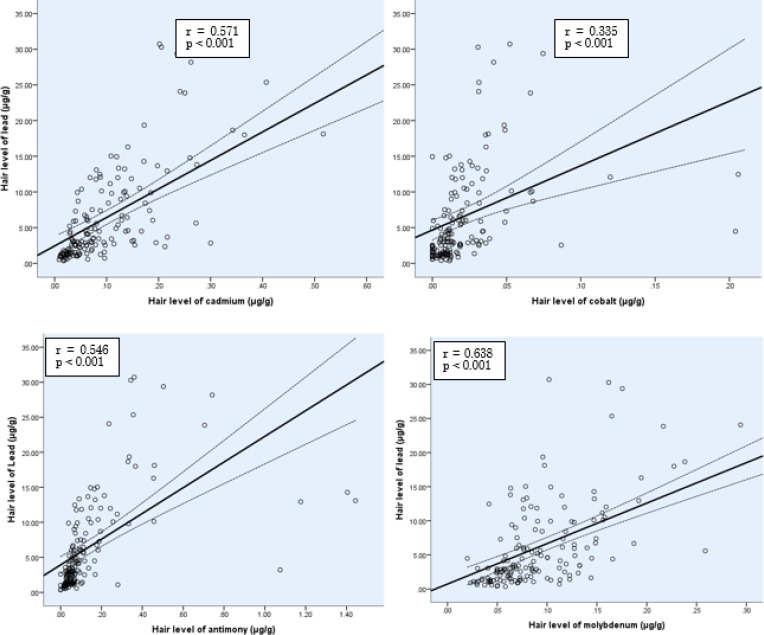
Pearson correlation between concentrations of lead with metals in children hair

There was a significant negative correlation between lead and zinc in children’s hair (r=蜢0.436, *P*<0.001) (Fig. 1). However, lead showed significant positive correlations with potentially toxic metals (cadmium, antimony, cobalt, and molybdenum) in children’s hair (Fig. 2).

Among 22 districts of Tehran, the most of study participants live in the district 17 (southwest, n=32) and the district 4 (northeast, n=29). There was not a significant difference between the children scalp hair metals levels or children’s weight with the district of living in the city (data not shown).

## Discussion

The present study found that relatively lower-weight children had higher levels of cobalt in their hair than children who weighed more at 18 months old. However, this difference disappeared when we adjusted for other variables in multiple regression analysis.

Children’s hair levels of some potentially toxic metals were approximately twice as high in girls as boys. Overall, girls also displayed lower weight than boys (Table 2). Although, the present study multivariate statistical analysis failed to demonstrate finding about cobalt and children weight, other study showed the content of metals in body fluids differences between the short stature children and the healthy children (25).

In the other word, gender influencing was reported on metals effect on body weight and/or body mass index (26). For instance, longitudinal studies in Bangladesh have shown that exposure to inorganic arsenic through drinking water during pregnancy and the postnatal period (measured in urine at 18 months) is correlated with decreased size at birth (27) and a lower childhood growth rate (at 18–24 months) (28), and the effect is more pronounced in girls than in boys. Similar findings regarding gender were observed in China, when comparing children based on arsenic levels in their drinking water (2 μg/L arsenic content vs. 190 μg/L) (29). Thus, gender may play an important role in metal toxicokinetics and accumulation, and consequently, in metal toxici-ties in children (30–32).

As the current study results, accumulation of toxic metals was showed (33–34) vary according to participants’ gender (36–37). An Italian study on children (aged 11–13 yr) reported higher levels of many elements (cadmium, cobalt, copper, zinc, and so on) in scalp hair of girls than in boys (38). In addition, many studies have revealed higher blood levels of manganese, copper, arsenic, cadmium, and selenium in females than males (39–41). Similar to hair and blood, a study has shown increased level of urinary cadmium in females than in males (35). On the other hand, some studies on children have reported no gender difference (42) or even lower blood levels of metal (lead) in girls than in boys (36, 43, 44). It is difficult to explain why metals levels were higher in girls than boys. However, it could be related to female vulnerability to absorb metals and/or a sex-related metabolic difference of metals (45), such as a higher bone release of metals (lead) in exchange with calcium, in female (46). In average, girls have a shorter growth period than boys due to complete their skeletal growth generally earlier and faster than boys (33, 39, 40).

In the present study, zinc showed a significant negative correlation with lead. Similar findings (in hair and blood) have been also reported in earlier studies (47–49). Perhaps, there is a competition between lead and zinc from absorption to the site of effects, as co-administration of zinc supplements and lead in experimental study revealed a reduction in lead absorption and accumulation in organs (50–53). Therefore, zinc may have a protective effect against lead, mediating by competition to various binding sites (54), such as metal-binding proteins (e.g., metallothioneins) (55), and zinc allosteric sites (56).

The sources of metal exposure for the study participants were not known. However, hair concentrations of metals in the children may be affected by biological, personal, and environmental factors (57). Among these, environmental pollution (mainly anthropogenic) could be the main source of exposure in Tehran, as the city has considerable numbers of industrial sources. Although concentrations of many elements are lower in residential zones than in industrial areas, some elements are present in the same (arsenic and zinc) or even higher levels (manganese, antimony, and copper) in these zones (18). Similarly, children who live near a high traffic road were exposed to higher levels of metals than those who did not (58), but the present study information about participants’ inhabitation did not include distance from roads. Some heavy metal contents were reported to be higher along the city roads (i.e., cadmium, 4 mg/kg; lead, 669 mg/kg; zinc, 614 mg/kg) (19) than the acceptable levels in natural soils (cadmium, 3 mg/kg; lead, 100 mg/kg; zinc, 300 mg/kg) (59). Therefore, the study participants may be exposed to potentially toxic metals mainly via industrial activities and vehicle exhaust fumes.

There were some limitations of the current study. First, the children’s hair samples were collected at different ages (20–36 months old), while weight was taken from medical records from birth to 18 months old. Second, we did not wash the hair samples before analysis. Third, we had limited information on the children’s diets and the fathers’ anthropometric characteristics. The mothers were asked to provide details of the fathers’ anthropometric characteristics, but the data were not sufficiently reliable to include in the statistical analyses. Finally, the current study did not measure metal levels in the tap water or in the air of Tehran.

## Conclusion

Children with relatively low weight gain had higher level of cobalt, which the finding disappeared when adjusting for covariates. In addition, girls showed higher levels of toxic metals than in boys. This may suggest that gender plays an important role in the absorption and/or accumulation of metals. The study also revealed significant correlations among measured metals in the children scalp hair. Thus, the future studies should be conducted on mix metals set and should consider gender as an important factor in metals toxicity.

## Ethical considerations

Ethical issues (Including plagiarism, informed consent, misconduct, data fabrication and/or falsification, double publication and/or submission, redundancy, etc.) have been completely observed by the authors.
